# The Patent Rubber

**Published:** 1863-03

**Authors:** 


### Editorial.

### THE PATENT RUBBER.

We have frequent inquiries in reference to this subject, more than we have time to answer by the pen, and hence we venture a few suggestions through this medium, yet with a feeling that we are incompetent to advise, and scarcely feel safe in making suggestions, for we know very little in reference to the matter.

The patent in reference to Hard Rubber originated entirely outside of the Dental profession, and only within the last few years has the profession become in any way connected with the matter, and even then, not in such a way as to elicit the special attention and solicitude of those most interested in the development and progress of the profession.

In regard to the validity of this patent, we have but little to say, because we do not know much about it, neither have we much solicitude in the matter. We presume, however, that the patent is as valid as most other patents; and if it covers the vulcanization of all other articles made of Hard Rubber, it of course will the base for artificial teeth.

It is declared that the decisions in the recent suits against parties manufacturing common articles of Hard Rubber, settle the matter, fully establishing the validity of the patent.

If dentists may go on, and indiscriminately employ hard rubber, then any other parties who may desire to do so, may go on and manufacture any articles whatever, regardless of the patent.

We have often said that others have the same opportunity of judging of the validity of this patent as ourselves. Our personal feelings are against patents in the profession, but this is an outside patent, one that comes into the profession incidentally and in order that the patent be valid any where, it must be protected. If the dental profession may use it with impunity, and justly, without grant, then every body else may do the same thing, and hence it is necessary to protect the interest of the patentee here, or give it up every where. Could the dentist take up any other outside patent, take for instance that of any patented machinery, and use it in the exercise of his profession, would it be just, or would he expect to use it in defiance of grant or license? Nevertheless we are opposed to all patents that originate in the profession, with professional men, as such.

And now as to what dentists should do in this matter, we hardly feel like suggesting; one course, however, they can pursue that will place them upon the safe and easy side. The Rubber Company have been disposed to be very lenient toward the Dental profession, at least they have instituted very few prosecutions. The use of rubber is certainly a good thing, of great value in the Dental profession, and one that we would hardly know how to do without, notwithstanding it has been the means of much mischief, of which we propose to speak hereafter. It may, perhaps, be made a matter of economy both to the dentist and his patient, and perhaps we can well afford to pay something for the use.

We would suggest to the company, however, that they should protect those who purchase their license.

The course which hard rubber has taken in the dental profession, demonstrates pretty clearly that any thing that is of real value in the profession can not very easily be hedged up, and made absolutely exclusive, by patent rights. T.

